# MicroRNA-21 regulate the cell apoptosis and cell proliferation of polycystic ovary syndrome (PCOS) granulosa cells through target toll like receptor TLR8

**DOI:** 10.1080/21655979.2021.1969193

**Published:** 2021-09-13

**Authors:** Yingying Yu, Guojing Li, Xiaoying He, Yu Lin, Zheng Chen, Xianhua Lin, Hong Xu

**Affiliations:** a Department of Obstetrics and Gynecology, International Peace Maternity and Child Health Hospital, School of Medicine Shanghai Jiao Tong University, Shanghai, China; b Shanghai Key Laboratory of Embryo Original Diseases, Shanghai, China; c Shanghai Municipal Key Clinical Specialty, Shanghai, China

**Keywords:** miR-21, PCOS, granulosa cell, TLR8

## Abstract

Polycystic ovary syndrome (PCOS) is a complex reproductive endocrine disease characterized by polycystic ovary. The aim of the study was to assess microRNA-21 regulates granulosa cell apoptosis and proliferation in polycystic ovary syndrome through target toll-like receptor 8. Granulosa cells were collected from 30 PCOS patients and 30 normal patients with tubal or male factor infertility (control) during in vitro fertilization-Embryo Transfer (IVF-ET) and were flash frozen with liquid nitrogen for storage for subsequent use. PCOS diagnosis was based on the revised standards of the American Society of Reproductive Medicine (ASRM) and the Rotterdam criteria PCOS granulosa cells and control granulosa cells were cultured in DMEM/F12 medium containing 10% fetal bovine serum and 1% antibiotic. After this RT-PCR, Western blot assessment and Detection of apoptosis by flow cytometry were conducted. The results of qPCR showed that the mRNA and protein expression of TLR8 in PCOS granulosa cells were significantly increased compared with the normal group. The results of Western blot also showed that the expression of TLR8, IFN-γ, TNF-α and IL-12 gene protein in the transfected cells was significantly higher than that in the control cells. Here, we show that miR-21 and TLR8 significantly increased in PCOS granulosa cell as compared with normal granulosa cells, and miR-21 enhances the TLR8 mRNA translation and then promotes the IFN-γ, TNF-α, and IL-12 secretion. Our study demonstrates that miR-21/ TLR8 involved in the PCOS inflammation, it provides profound insights into pathogenesis of PCOS.

## 1. Introduction

PCOS is defined by a combination of signs and symptoms of androgen excess and ovarian dysfunction in the absence of other specific diagnoses. PCOS is frequently associated with abdominal adiposity, insulin resistance, obesity, metabolic disorders, and cardiovascular risk factors [1] Polycystic ovary syndrome (PCOS) is a common reproductive endocrine disorder and metabolic disorder in women of childbearing age 5%–10% [1]. A wide range of pathophysiological changes are related to neuroendocrine, immunology, protein metabolism, sugar metabolism, fat metabolism, and abnormal local ovarian regulatory factors [2].

The diagnosis and treatment of PCOS are not complicated, requiring only the judicious application of a few well-standardized diagnostic methods and appropriate therapeutic approaches addressing hyperandrogenism, the consequences of ovarian dysfunction and the associated metabolic disorders [3].

In recent years, it was found that the levels of inflammatory marker factors (such as C-reactive protein, calcitonin, tumor necrosis factor, IL-6, IL-18.) in peripheral blood of patients with PCOS were significantly higher than those in the control group, suggesting that PCOS may be a chronic inflammatory reactive disease [3].

Inflammation is usually caused by microbial infection, the toll-like receptor can recognize the inflammatory response induced by invasive pathogens, activate the downstream signal pathway, and promote the expression of pro-inflammatory factor protein gene [4,5]. Toll-like receptor (TLR) is a member of pattern recognition receptor (PRRS) family, which plays an important role in innate immunity and adaptive immunity. TLR is classified by recognizing different ligands. At present, the main human-related receptors are TLR1-TLR10 [6], widely expressed in cells of innate immune system and adaptive immune system, and expressed in macrophages, epithelial cells, endothelial cells, parenchymal organ cells, T cells, B cells, mast cells, and other cells. Recently, it has been found that endogenous cytokines released from tissue injury and endogenous molecules from saturated fatty acids can activate TLR [7], which indicates that TLR not only mediates the inflammatory response caused by infection but also mediates the inflammation induced by endogenous molecules.

More strikingly, recent studies indicate that miRNAs can even serve as physiological ligands for toll-like receptors (TLRs), a function that is independent of their conventional role in post-transcriptional gene regulation [7,8].

It has been found that the expression levels of TLR2, TLR8, and CD14 in PCOS patients’ samples increased significantly through functional and signal pathway enrichment analysis, and they also enriched significantly in inflammatory and immune-related signal pathways [8]. TLR8, as a member of TLRs family, is mainly located in the cytoplasm and expressed in monocytes, macrophages, and myeloid dendritic cells [9]. It can recognize the nucleic acids of bacteria or viruses [10] and plays an important role in innate immune response, cell signal transduction, neuron development, and cell apoptosis. At present, a large number of literatures have reported that TLR7, TLR8, and TLR9 are highly expressed in many autoimmune and infectious diseases, such as discoid lupus erythematosus, rheumatoid arthritis, psoriasis, and so on [11].

The interaction between miR-21/29a and TLR7/8 may cause macrophages to secrete proinflammatory cytokines (e.g., TNF-α and IL-6), ultimately leading to tumor growth and metastasis [12,13]. Thus, by acting as paracrine ligands of TLRs, miRNAs are key regulators of the tumor microenvironment. This mechanism of action of miRNAs is implicated in tumor-immune system communication and is important in tumor growth and spread, thus representing a possible target for cancer treatment [14]. Studies have shown that TLR8 activation leads to the secretion of inflammatory cytokines such as IFN-γ, TNF-α, and IL-12.

The discovery of this new function of miRNAs raises some fascinating questions. First, what is the underlying mechanism through which miRNAs bind to intracellular TLRs? According to the current results, the GU-rich motif (GUUG for miR-21, GGUU for miR-29a, and GUUGUGU for let-7b) is essential for the miRNA-TLR recognition. Thus, individual miRNAs may contain important information that allows selective access of these miRNAs to TLRs [14,15].

Muller [16] found that human tumor cells can secrete miR-21 and miR-29a, which bind to TLR8 in immune cells and activate TLR8, leading to activation of NF-κB and secretion of pro-inflammatory cytokines. MiR-21 is a member of miRNAs, which can regulate multiple target genes through 3ʹUTR. The end complementary combination can degrade or block the translation of the target gene, lead to the abnormal expression of the target gene, regulate the biological functions such as cell proliferation, migration, and cycle, and play a role in the occurrence, development, and metastasis of various tumors, which is closely related to multiple signaling pathways of human life activities. The aim of the study was to assess microRNA-21 regulates granulosa cell apoptosis and proliferation in polycystic ovary syndrome through target toll-like receptor 8.

## 2. Material and methods

MiR-21 MICs was synthesized by Shanghai Biotechnology Co. Ltd, Plasmid Extraction Kit; Lipofectamine 2000 and hygromycin were purchased from Invitrogen company; DMEM/F12 medium, fetal bovine serum, and antibiotics were purchased from GIBCO company; reverse transcription kit was purchased from Takara company; SYBR Green I PCR master mix was purchased from Toyobo company; CCK-8 kit was purchased from Japan Tongren Research Institute; annexin v Fitc PI staining and apoptosis detection kit was purchased from BD company, Abcam company, and Zhongshan Jinqiao company, respectively.

### 2.1. Cell culture

Granulosa cells were collected from 30 PCOS patients and 30 normal patients with tubal or male factor infertility (control) during in vitro fertilization-Embryo Transfer (IVF-ET) and were flash frozen with liquid nitrogen for storage for subsequent use. The study protocol was approved by the Ethics Committee of the International Peace Maternity and Child Health Hospital, and was initiated after obtaining signed informed consent from all participants. Patients for the PCOS and control groups were selected according to the inclusion and exclusion criteria laid out below.

PCOS diagnosis was based on the revised standards of the American Society of Reproductive Medicine (ASRM) and the Rotterdam criteria [18]. Satisfaction of any 2 of the 3 criteria constitutes a positive PCOS diagnosis and excludes other hyperandrogenic disorders: 1) no ovulation or rare ovulation; 2) clinical and biochemical changes in androgen levels; 3) polycystic ovarian changes. China and was initiated after obtaining signed informed consent from all participants.

PCOS granulosa cells and control granulosa cells were cultured in DMEM/F12 medium containing 10% fetal bovine serum and 1% antibiotic; HEK293T cells were cultured in DMEM medium containing 10% fetal bovine serum and 1% antibiotic, all of them were placed in 37°C, 5% CO2 incubator, when 80% of them were fused, digested with 0.25% trypsin, and inoculated into the culture plate with DMEM/F12 or DMEM complete medium, and continue to cultivate.

### 2.2 RNA extraction and QRT PCR

Total RNA was extracted with TRIzol (Invitrogen, USA). The total RNA of Control Granular cells, PCOS Granular cells, and PCOS-miR-21 mimics cells and PCOS-miR-21 mimics NC cells which transfected for 48 hours. The total RNA was transformed into cDNA by reverse transcription kit. The reaction conditions were 37°C, 15 min, 85°C, 5 s. Then, PCR amplification was carried out with the cDNA as the template. Reaction conditions: 95°C, 10 min, 95°C, 20 s, 60°C, 30 s, 72°C, 40 s, 45 cycles. With GAPDH as a reference, the relative expression of TLR8, IFN -γ, TNF-α and IL-12 genes was calculated by comparative CT method. The formula was 2-ΔΔCT.

### 2.3. Western blot

Collected control granular cells, PCOS granular cells, and collected PCOS-miR-21 mimics cells and PCOS-miR-21 mimics NC cells, which transfected for 48 hours. The total protein was extracted by cell lysate, and then the total protein was electrophoresis by SDS-PAGE. After electrophoresis, the gel was transferred to PVDF membrane. The TBST (Shanghai Shenggong Biological Co., Ltd., China) containing 5% skimmed milk powder was sealed at room temperature for 2 hours. The first antibody (TLR8 1:500, IFN-γ 1:1000, TNF-α 1:1000 and IL-12 1:1000, GAPDH 1:10,000) was added. The TBST was incubated overnight at 4°C. The TBST was washed three times, each time for 5 min. The second antibody was incubated at room temperature for 2 hours. The chemiluminescence method was used for color development for 3 min, and the film was developed by pressing.

### 2.4. Detection of difluorinase reporter gene

The wild type (WT) and mutant (MUT) recombinant reporter gene plasmids of TLR8 were synthesized by Genecopoeia from Guangzhou, China. These plasmids were cotransfected into HEK293T cells with miR-21 mimic and mimic control by using x-tremegene siRNA transfection reagent. After 48 hours of transfection, the culture supernatant was collected and passed through the secret pair according to the manufacturer’s instructions. The detection was performed by genecopoeia.

### 2.5. CCK-8 colorimetry for cell proliferation rate

According to the instructions of CCK-8 reagent, take the PCOS granulosa cells of logarithmic growth period, inoculate them in 96 well plate with 5 × 103 cell density and 100 μl per well. Experimental groups: blank control group (non-transfected group), negative control group (transfected with miR-21 mimics NC group), and experimental group (transfected with miR-21 mimics group). After 48 hours, cells in each group were collected, added with CCK-8 reagent with final concentration of 10%, incubated in 37°C incubator for 100 min, and then the absorbance of 450 nm was measured on the enzyme scale. The average of three values of each group was measured, and the proliferation rate was calculated, and the proliferation rate curve was drawn Proliferation rate (%) = (experimental group a blank control group)/(negative control group a blank control group) 100%. The experiment was repeated three times.

### 2.6. Detection of apoptosis by flow cytometry

PCOS-miR-21 mimics and PCOS-miR-21 mimics NC cells transfected for 48 hours were collected and washed with precooled PBS. 500-μl binding buffer was added to each group to resuspend the cells at a density of 1 × 106 /ml, and 5 μ l annexin V FITC was added to each group. The gently mix cells and dyes were incubated in dark and normal temperature for 15 min, then 1 × 104 cells were detected by flow cytometry within 1 hour. Cell quest was used to analyze the apoptosis of three groups of cells. The experiment was repeated three times.

### 2.7. Cell morphology observation

Traditional HE staining method was used to fix the cell smear in 95% ethanol for 15 min. Under the microscope, the immunostaining area was selected and marked. Xylene was used to dewax the staining. After the section was cleaned, the resin was washed with ethanol and distilled water gum. Hematoxylin staining solution was used for 3–5 min, 0.5% dilute hydrochloric acid solution was used for cleaning, rinse with water and soak for 15 min, then seal with gum or DPX cover.

### 2.8. Statistical treatment

SPSS17.0 statistical software was used to analyze the data. The measurement data were expressed by mean ± standard deviation (SD). Single factor analysis of variance was used to compare multiple groups of data. Student-Newman-Keuls (SNK)-q test was used to compare the mean of each concentration group. Both sides a = 0.05, the difference was statistically significant (*P* < 0.05).

## 3. Results

The results of the study revealed that mRNA and protein expression of TLR8 in PCOS granulosa cells were significantly increased. Real-time fluorescent quantitative PCR showed that the mRNA expression levels of TLR8, IFN-γ, TNF-α and IL-12 genes were significantly higher in test group. Total apoptosis rate of PCOS particles in miR-21 mimics group was significantly lower than that in NC group.

### 3.1. High expression of TLR8 and miR-21 in PCOS ovarian granulosa cells

**Figure 1.**
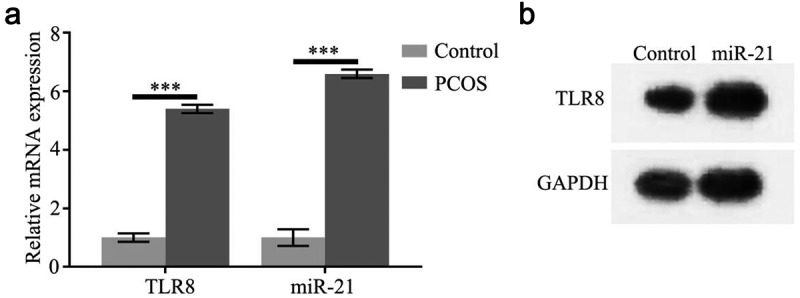
High expression of TLR8 and miR-21 in PCOS ovarian granulosa cells

The results of qPCR showed that the mRNA and protein expression of TLR8 in PCOS granulosa cells were significantly increased compared with the normal group (Figure 1). The mRNA expression of miR-21 in PCOS granulosa cells was significantly increased compared with the normal group (Figure 1(a)).

(a) Relative mRNA expression of TLR8 and miR-21 in PCOS ovarian granulosa cells, (b) Relative protein expression of TLR8 in PCOS ovarian granulosa cells.

### 3.2. Transfection with miR-21 decreased the luciferase activities of the TLR8 3ʹ-UTR but not the mutant one

**Figure 2.**
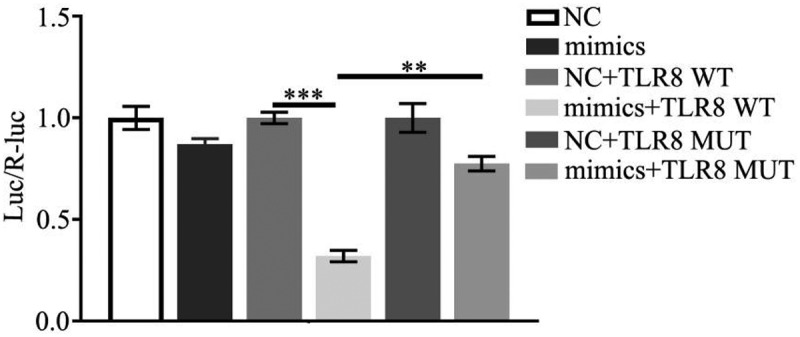
Binding verification of miR-21 and TLR8 by double luciferase reporter gene

The results of double luciferase reporter gene assay showed that there was no significant difference in luciferase activity compared with miR-21 transfected with TLR8 3ʹUTR mutant vector plasmid. By transfection of TLR8 3ʹUTR wild-type vector, the luciferase activity of miR-21 group was significantly inhibited (Figure 2).

### 3.3. Detection of TLR8, IFN-γ, TNF-α, and IL-12 gene expression after over expression of miR-21

**Figure 3.**
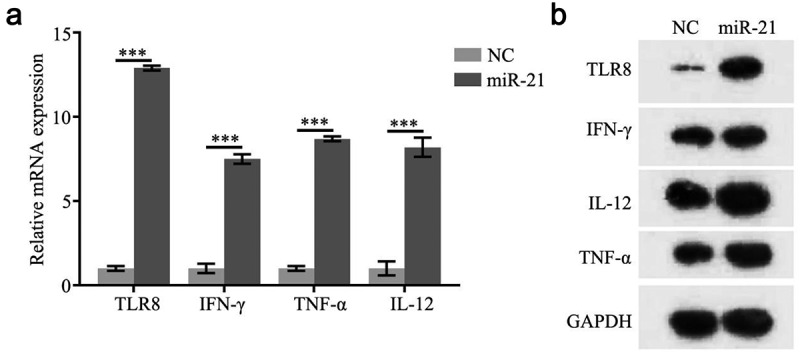
miR-21 regulate the expression of TLR8, IFN-γ,TNF-α, and IL-12

The expression of TLR8, IFN-γ, TNF-α, and IL-12 genes was detected by total RNA and total protein of lysed cells after transfected miR-21 mimics and miR-21 mimics NC into PCOS granulosa cells 48 hours. The results of real-time fluorescent quantitative PCR showed that as PCOS group as compared with the control group, the mRNA expression levels of TLR8, IFN-γ, TNF-α, and IL-12 genes were significantly higher (Figure 3(a)). The results of Western blot also showed that the expression of TLR8, IFN-γ, TNF-α, and IL-12 gene protein in the transfected cells was significantly higher than that in the control cells (Figure 3(b)).

(a) Relative mRNA expression of TLR8, IFN-γ, TNF-α, and IL-12, (b) Protein expression of TLR8, IFN-γ, TNF-α, and IL-12.

### 3.4. The effect of miR-21 on the cell apoptosis and cell proliferation of PCOS granulosa cells

**Figure 4.**
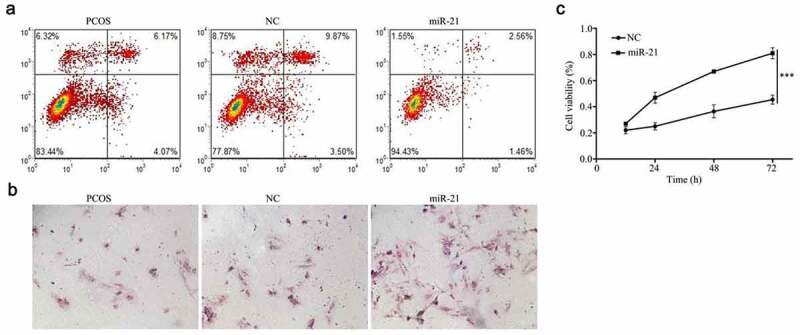
The effect of miR-21 on the cell apoptosis and cell proliferation of PCOS granulosa cells

After expressing miR-21, the total apoptosis rate of PCOS particles in miR-21 mimics group (38.73 ± 2.27)% was significantly lower than that in NC group (8.84 ± 1.21)% and untransfected group (15.16 ± 1.82)%, the difference was statistically significant (F = 64.48, *P* < 0.001), but there was no significant difference between the untransfected group and the empty vector control group (Figure 4(a)). Compared with NC group, the proliferation of PCOS granulosa cells increased after miR-21 mimics was transfected, and with the increase of time, the corresponding cell proliferation rate increased, that is, time-dependent. There was significant difference in the proliferation rate among the groups (Figure 4(c)). The staining results HE PCOS ovarian granulosa cells showed that the cell density increased and the morphology changed after overexpression of miR-21 compared with that of untransfected group and transfected empty vector group (Figure 4(b)).

(a) the cell apoptosis of PCOS granulosa cells, (b) HE staining images of PCOS granulosa cells, (c) The cell proliferation of PCOS granulosa cells.

## 4. Discussion

The results of study showed that the mRNA and protein expression of TLR8 in PCOS granulosa cells were significantly increased compared with the normal group.

It is well known that PCOS is characterized by ovarian dysfunction, hyperandrogenemia, IR, and hyperinsulinemia [18]. More and more literatures analyzed the influencing factors of PCOS from the perspectives of endocrine, immunology, gene genetics, and molecular biology. All other research studies also showed same results and concluded that the levels of inflammatory marker factors (such as C-reactive protein, calcitonin, tumor necrosis factor, IL-6, IL-18.) in peripheral blood of PCOS patients were significantly higher than those of the control group, suggesting that PCOS may be a chronic inflammatory reactive disease [15]. So authors of the study found that the PCOS patients not only have abnormal endocrine metabolism but also having chronic inflammation.

The results of double luciferase reporter gene assay showed that there was no significant difference in luciferase activity compared with miR-21 transfected with TLR8 3ʹUTR mutant vector plasmid. The other studies also revealed the same findings.

miRNA is a kind of naturally occurring non-coding microRNA (ncRNA) with about 22 nucleotides [19]. More and more studies have shown the influence of miRNA on biological fields, such as development, differentiation, cell proliferation, apoptosis, metabolism, inflammatory response, and various diseases [20]. In mammalian systems, recent studies have shown that miRNAs regulate TLR signaling pathways at different levels, including TLRs, signaling proteins, transcription factors, cytokines, etc [21]. As we all know, miRNA binds to the complementary sites on the target mRNA through base pairing, which leads to translation inhibition or direct degradation of mRNA. However, recent studies have shown that miRNA can not only regulate gene transcription but also secrete into extracellular environment as signal molecules that mediate intercellular communication [22], or transport from cytoplasm to nucleus to regulate their own expression [23]. MiRNAs can even be used as physiological ligands of TLRs, and their functions are independent of their regulatory genes [16]. TLRs in cells including TLR3, TLR7, TLR8, and TLR9 can detect foreign nucleic acids, including RNA from RNA viruses and DNA from bacteria and DNA viruses [24]. All the above results indicate that there is a certain relationship between miRNA and TLR signaling pathway. For TLR8, some studies have shown that miR-21 and miR-29 may secrete exosomes from tumor cells to immune cells, and combine with TLR8 to regulate intracellular signaling pathway [17].

The results of real-time fluorescent quantitative PCR showed that compared with the control group, the mRNA expression levels of TLR8, IFN-γ, TNF-α, and IL-12 genes were significantly higher. The results of Western blot also showed that the expression of TLR8, IFN-γ, TNF-α, and IL-12 gene protein in the transfected cells was significantly higher than that in the control cells. . TLR-8 can be activated by miR-21, which leads to the secretion of IFN-γ, TNF-α, and IL-12. The expression of IFN-γ, TNF-α, and IL-12 in PCOS granulosa cells is significantly increased, which proves that TLR-8 is involved in the development of PCOS inflammation. MiR-21 is a member of miRNAs, which can regulate multiple target genes through 3ʹUTR. The end complementary combination can degrade or block the translation of the target gene, lead to the abnormal expression of the target gene [17,25], regulate the biological functions such as cell proliferation, migration and cycle, and play a role in the occurrence, development, and metastasis of various tumors, which is closely related to multiple signaling pathways of human life activities. In our study, compared with NC group, the proliferation of PCOS granulosa cells increased after miR-21 mimics was transfected, and with the increase of time, the corresponding cell proliferation rate increased, that is, time-dependent. There was significant difference in the proliferation rate among the groups. The staining results HE PCOS ovarian granulosa cells showed that the cell density increased and the morphology changed after overexpression of miR-21 compared with that of untransfected group and transfected empty vector group. **So**, after overexpression of miR-21, the proliferation rate of PCOS granulosa cells increased significantly, while the apoptosis rate decreased. It is proved that miR-21 can regulate cell proliferation, migration, cycle and other biological functions, and play a role in the occurrence, development and metastasis of various tumors.

Conclusion: miR-21/ TLR8 involved in the PCOS inflammation, it provides profound insights into pathogenesis of PCOS. TLRs activate NF-κB, MAPK signaling pathway, release NF-κB, IFN – α, and other factors, up regulate the expression of costimulatory molecules on the surface of antigen-presenting cells, initiate natural immune response, activate nonspecific immune response, participate in the process of immune defense, and play an important role in the formation and development of inflammation and tumor. The molecular mechanism of miR-21/TLR8 signaling pathway regulates which target genes affect the genesis and development of PCOS ovarian granulosa cells is the focus of our future research.

## Highlights

1. mRNA and protein expression of TLR8 in PCOS granulosa cells was significantly increased.
2. Real-time fluorescent quantitative PCR showed that the mRNA expression levels of TLR8, IFN-γ, TNF-α, and IL-12 genes were significantly higher in test group.
3. Total apoptosis rate of PCOS particles in miR-21 mimics group was significantly lower than that in NC group.
